# Neuroprotective Role of E3 Ubiquitin Ligase TRIM2 in Parkinson's Disease: Attenuation of Oxidative Stress and Apoptosis via Promoting ELAVL1 Ubiquitination

**DOI:** 10.1002/cns.71075

**Published:** 2026-08-12

**Authors:** Wei Liang, WenJie Sun, ZhiJun Zhao, Ke Song, JinYing Jia

**Affiliations:** ^1^ Department of Integrated Traditional Chinese and Western Medicine The First Affiliated Hospital of Zhengzhou University Zhengzhou China; ^2^ Traditional Chinese Medicine Integrated Department of Nephrology The First Affiliated Hospital of Zhengzhou University Zhengzhou China

**Keywords:** ELAVL1, parkinson's disease, TRIM2, ubiquitination

## Abstract

**Background:**

Parkinson's disease (PD) is characterized by the progressive loss of dopaminergic neurons, where oxidative stress and neuronal apoptosis are key pathogenic events. In this study, we identified a downregulated TRIM2 in the substantia nigra pars compacta (SNc) of PD rats based on label‐free proteomics. However, the impact of TRIM2 on PD is unknown.

**Methods:**

We used 6‐hydroxydopamine (6‐OHDA) to construct a PD model in vivo and in vitro.

**Results:**

TRIM2 overexpression alleviated neurobehavioral deficits, mitigated the loss of dopaminergic neurons, and suppressed oxidative stress and apoptosis in the SNc of PD rats. These effects were also observed in the 6‐OHDA‐treated differentiated BE (2)‐M17 cells. Mechanistically, the RNA‐binding protein ELAVL1 was identified as a critical downstream target, given that the database predicts it to be a TRIM2‐interacted protein and a PD‐related protein. Herein, TRIM2 directly interacted with ELAVL1 and promoted its ubiquitin‐mediated degradation. Crucially, rescue experiments confirmed that the neuroprotection conferred by TRIM2 was counteracted by ELAVL1 overexpression in the 6‐OHDA‐treated BE (2)‐M17 cells that exhibited neuronal‐like properties.

**Conclusion:**

Our findings uncovered a novel TRIM2‐ELAVL1 axis as a pivotal regulatory mechanism in PD pathogenesis, positioning TRIM2 as a potential target for therapeutic intervention in PD.

## Graphical abstract

The E3 ubiquitin ligase TRIM2 confers neuroprotection against 6‐OHDA‐induced oxidative stress and apoptosis. It exerts this function by binding to ELAVL1 and promoting its degradation via the ubiquitin‐proteasome pathway. Thus, our study reveals the TRIM2‐ELAVL1 axis as a key regulatory mechanism for neuronal survival under 6‐OHDA‐stimulated toxic stress.

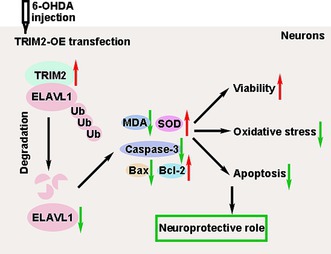

**Abbreviations**
6‐OHDA: 6‐hydroxydopamine
AAV2: Adeno‐associated virus type 2
ARE: Antioxidant response element
BIM: Bcl‐2‐interacting mediator of cell death
BP: Biological process
BSA: Bovine serum albumin
CC: Cellular component
CHX: Cycloheximide
Co‐IP: Co‐immunoprecipitation
ELAVL1: ELAV‐like RNA‐binding protein 1
GO: Gene Ontology
IF: Immunofluorescence
IHC: Immunohistochemistry
KEGG: Kyoto Encyclopedia of Genes and Genomes
LDH: Lactate dehydrogenase
MDA: Malondialdehyde
MF: Molecular function
MPP^+^
: 1‐methyl‐4‐phenylpyridinium
NLRP3: NACHT, LRR and PYD domains‐containing protein 3
NRF2: Nuclear factor erythroid 2‐related factor 2
PCA: Principal component analysis
PD: Parkinson's disease
PVDF: Polyvinylidene fluoride
RA: Trans‐retinoic acid
SD: Sprague–Dawley
SDS‐PAGE: Sodium dodecyl sulfate‐polyacrylamide gel electrophoresis
SNc: Substantia nigra pars compacta
SOD: Superoxide dismutase
TH: Tyrosine hydroxylase
TRIM: Tripartite motif
XIAP: X‐linked inhibitor of apoptosis
α‐Syn: α‐synuclein

## 1 Introduction

Parkinson's disease (PD) represents a prevalent neurodegenerative disorder, pathologically characterized by the progressive loss of dopaminergic neurons in the substantia nigra pars compacta (SNc), which ultimately culminates in motor dysfunction [1]. Although several pharmacological agents, such as Levodopa and Amantadine, are currently available to alleviate the symptoms of PD, their therapeutic efficacy is often limited and may be accompanied by a spectrum of adverse effects [2]. Interestingly, oxidative stress has emerged as a pivotal contributor to PD progression [3, 4]. Consequently, the exploration of therapeutic targets, especially those with antioxidant properties, holds significant promise for developing novel interventions for PD.

E3 ubiquitin ligases are pivotal enzymes within the ubiquitin‐proteasome system, orchestrating the specific degradation of target proteins by recognizing substrates and facilitating ubiquitin transfer to lysine residues. Among them, the RING‐type E3 ubiquitin ligases constitute the largest subclass [5, 6]. The tripartite motif (TRIM) family represents a major subgroup of RING E3 ligases, whose members are implicated in diverse cellular processes, including inflammation [7], innate immunity [8], oncogenesis [9], and neurodegenerative disorders [10]. Herein, we established a 6‐hydroxydopamine (6‐OHDA)‐induced rat PD model and employed label‐free proteomics to identify the proteins that were differentially expressed in the SNc of the 6‐OHDA rats compared with healthy controls (Fold Change > 1.5 or < 0.67, *p* < 0.05). We noted two TRIM family members, TRIM2 and TRIM32. A previous study has indicated that TRIM32 downregulates X‐linked inhibitor of apoptosis (XIAP), thereby promoting neuronal death under PD stress conditions [11]. However, the role of TRIM2 in PD remains unexplored. We therefore focus here on TRIM2.

TRIM2 is expressed in the brain and predominantly localized in the neuronal cytoplasm. It contributes to the differentiation of neural progenitors [12]. TRIM2 deficiency leads to accumulation of the neurofilament light chain and neurodegeneration in mice [13]. TRIM2 suppression disrupts neuronal polarity, whereas TRIM2 overexpression stimulates axon specification through ubiquitination of the neurofilament light chain [14]. Consistently, loss‐of‐function mutations in TRIM2 are capable of causing axonal neuropathy [15, 16]. Furthermore, lentivirus‐mediated knockdown of TRIM2 stabilizes the pro‐apoptotic protein Bcl‐2‐interacting mediator of cell death (BIM), thereby attenuating neuroprotective function [17]. TRIM2 has been shown to confer protection against N‐methyl‐d‐aspartic acid‐induced apoptosis in retinal ganglion cells [18]. Notably, TRIM2 exerts oncogenic functions through activation of the ROS‐related nuclear factor erythroid 2‐related factor 2 (NRF2)/antioxidant response element (ARE) signaling pathway in pancreatic cancer [19]. The NRF2/ARE serves as a critical neuroprotective signaling pathway, defending against diverse oxidative stress‐related neurodegenerative insults and playing a substantial role in multiple neurodegenerative disorders, including PD [20, 21]. Furthermore, analysis of the GSE7621 dataset revealed a significant downregulation of TRIM2 in the substantia nigra of PD patients (Fold Change < 0.67, *p* < 0.05). Collectively, these findings prompt the potential significance of TRIM2 in PD pathogenesis and highlight the importance of further elucidating its downstream regulatory signals for the development of novel therapeutic strategies.

ELAV‐like RNA‐binding protein 1 (ELAVL1), also known as HuR, is predicted to potentially interact with TRIM2 and contains ubiquitination sites within its sequence based on the BioGrid database. A previous study has demonstrated that ELAVL1 expression promotion suppresses cell viability and accelerates apoptosis in the 1‐methyl‐4‐phenylpyridinium (MPP^+^)‐treated human neuroblastoma SK‐N‐SH cell line, a recognized in vitro PD model [22]. Importantly, loss of ELAVL1 reduces mitochondrial ROS production and cytotoxicity in MPP^+^‐treated SH‐SY5Y neuroblastoma cells [23]. These findings lead us to hypothesize that TRIM2 may modulate neuronal oxidative stress and apoptosis by regulating ELAVL1 expression, thereby influencing PD progression.

This study aims to investigate the impact of TRIM2 on oxidative stress and apoptosis in the PD model in vivo and in vitro and to explore its functional interaction with ELAVL1 in neuronal cells, thereby elucidating the neuroprotective role of the TRIM2/ELAVL1 axis in PD.

## 2 Materials and Methods

### 2.1 Animals

The details are provided in the Supporting Information. All animal experiments in this study were approved by The First Affiliated Hospital of Zhengzhou University (2025‐KY‐0297).

### 2.2 Label‐Free Proteomics

The details are provided in the Supporting Information.

### 2.3 Adeno‐Associated Virus Type 2 (AAV2) Particle Injection

Ten‐week‐old male SD rats were anesthetized and fixed in a stereotaxic apparatus. The cDNA encoding full‐length rat TRIM2 (NM_001108552) was cloned into an AAV2 vector under the control of the cytomegalovirus (CMV) promoter. Referencing previous literature, the AAV2‐mediated rat TRIM2 overexpressing vectors and empty vectors (2 μL) were injected slowly into the right substantia nigra of the rats at a dose of 1 × 10^11^ vg/ml [24] with an infusion rate of 1 μL/min (−5.3 mm anteroposterior, −1.8 mm mediolateral, and −8 mm dorsoventral relative to the bregma) [25]. Four weeks after AAV2 particle injection, the rats were intraperitoneally injected with 20 mg/kg imipramine and then injected with 6‐OHDA to establish a PD model as described above. After 28 days of 6‐OHDA injection, the rats were subjected to stepping and cylinder tests to evaluate motor function. Apomorphine‐induced rotational behavior tests were conducted after 28 and 32 days of 6‐OHDA injection. After the behavioral tests, the SNc tissues on the damaged side were collected and isolated for subsequent experiments.

### 2.4 Behavioral Observations

The details are provided in the Supporting Information.

### 2.5 Immunohistochemistry (IHC)

The details are provided in the Supporting Information.

### 2.6 Cell Culture and Treatment

BE (2)‐M17 cells (CBP60919M, CoBioer, Jiangsu, China) were purchased from Nanjing COBIOER BIOSCIENCES Co. Ltd. and cultured in MEM/F12 medium containing 10% fetal bovine serum at 37°C and 5% CO_2_ in an incubator (HF‐90, Shanghai Lishen, Shanghai, China).

The cells were treated with 10 μM trans‐retinoic acid (RA, R817255, Macklin, Shanghai, China) for 72 h to induce cell differentiation into a more salient neuronal cell type [26]. The differentiated cells were transfected with the plasmids for 48 h, and then the cells were subjected to a treatment of 80 μM 6‐OHDA (H873296, Macklin, Shanghai, China) for 18 h to establish a PD cell model according to previous literature [27]. The details of the plasmids are provided in the Supporting Information.

### 2.7 Cell Viability

Cell Counting Kit‐8 (BS350A, Biosharp, Beijing, China) kit was used to measure cell viability. The details are provided in the Supporting Information.

### 2.8 Lactate Dehydrogenase (LDH) Activity

The details are provided in the Supporting Information.

### 2.9 Caspase‐3 Activity

The details are provided in the Supporting Information.

### 2.10 Immunofluorescence (IF)

The details of IF staining are provided in the Supporting Information.

### 2.11 TUNEL

The details are provided in the Supporting Information.

### 2.12 Co‐IP

Co‐IP was performed in accordance with the Thermo Scientific Pierce immunoprecipitation kit (Thermo Fisher Scientific, USA). The details are provided in the Supporting Information.

### 2.13 Malondialdehyde (MDA) and Superoxide Dismutase (SOD)

The measurements of MDA content and SOD activity were conducted in accordance with the corresponding kits. The details are provided in the Supporting Information.

### 2.14 Real‐Time PCR

The details are provided in the Supporting Information.

### 2.15 Western Blot

The details are provided in the Supporting Information.

### 2.16 Statistical Analysis

Detailed statistical methods are provided in the Supporting Information.

## 3 Results

### 3.1 Downregulated TRIM2 Was Identified in the SNc of 6‐OHDA‐Lesioned PD Rat Model

**FIGURE 1:**
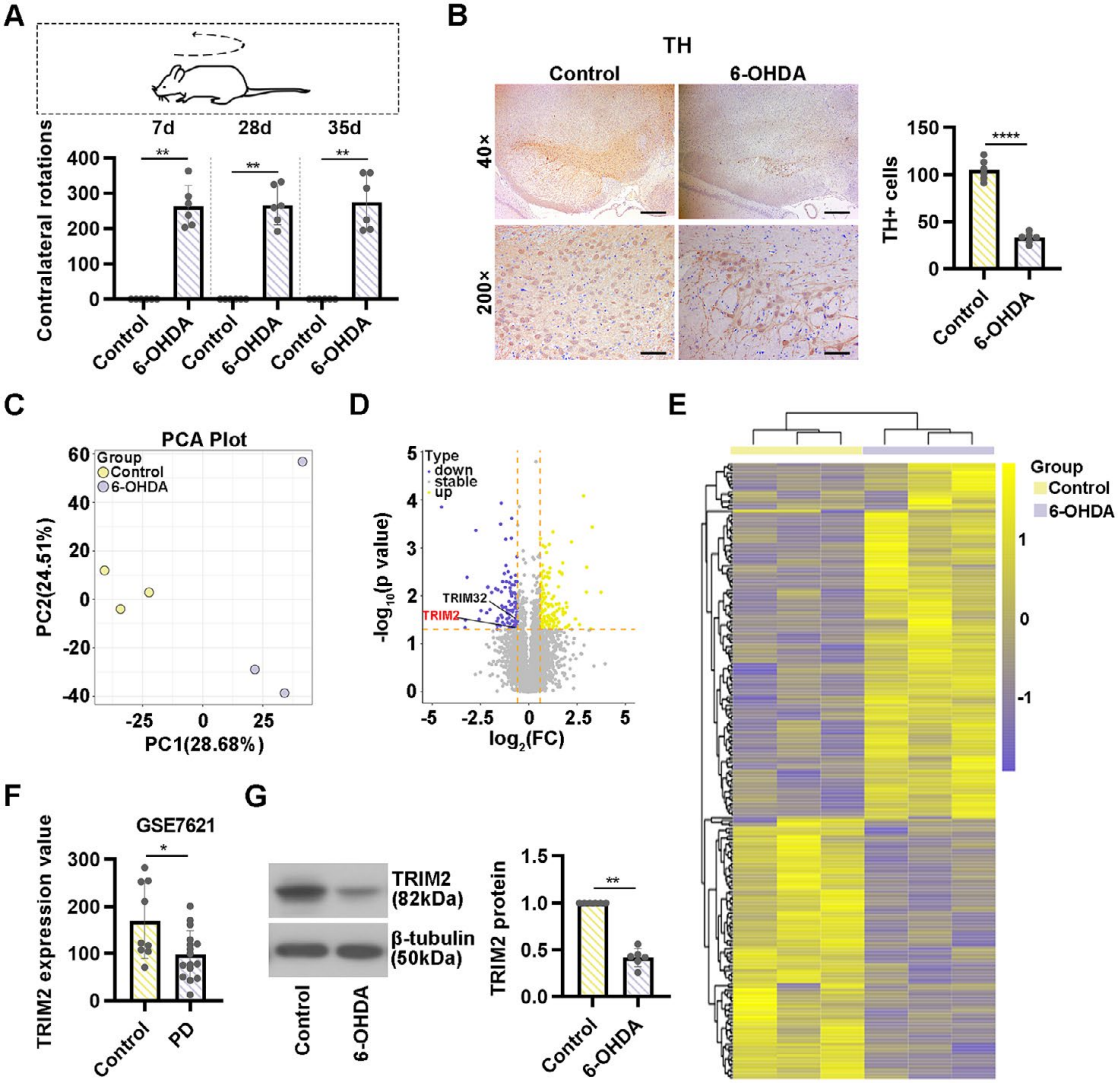
Downregulated TRIM2 protein was found in the substantia nigra pars compacta (SNc) of 6‐hydroxydopamine (6‐OHDA)‐induced Parkinson's disease (PD) rat model. (A) Apomorphine‐induced contralateral rotations at 7, 28, and 35 days following 6‐OHDA injection. (B) Validation of the PD model by tyrosine hydroxylase (TH) immunohistochemistry (IHC) in the rat SNc. 40×, scale bar = 500 μm; 200×, scale bar = 100 μm. *n* = 6 per group. (C) Principal component analysis (PCA) plot of control and 6‐OHDA groups based on proteomic sequencing. (D) Volcano plot of the differentially expressed proteins identified by proteomic sequencing (Fold Change > 1.5 or < 0.67, *p* < 0.05). TRIM family proteins among the differentially expressed proteins, TRIM2 and TRIM32, were marked. (E) Heatmap of the differentially expressed proteins. *n* = 3 per group. (F) Decreased TRIM2 expression in the substantia nigra tissues from 16 postmortem human brains of PD patients compared to 9 healthy controls based on the GSE7621 dataset. (G) Western blot further revealed a reduction in TRIM2 protein in the SNc of 6‐OHDA‐lesioned rats. *n* = 6 per group. *p < 0.05, **p < 0.01, ****p < 0.0001.

Apomorphine‐induced rotation behavior test confirmed that the number of contralateral rotations increased to over 100 within 30 min in the rats on the 7th, 28th, and 35th day after 6‐OHDA injection (Figure 1A, *p* < 0.01). Additionally, 6‐OHDA caused the reduction of TH‐positive neurons (dopaminergic neurons) in the rat SNc (Figure 1B, *p* < 0.0001). Proteomic sequencing was conducted to analyze the differentially expressed proteins in the 6‐OHDA‐treated rat SNc compared with the control tissues (Fold Change > 1.5 or < 0.67, *p* < 0.05). The principal component analysis (PCA) plot showed that there is a significant difference between 6‐OHDA and control samples (Figure 1C). The differentially expressed proteins were analyzed by Gene Ontology (GO) and Kyoto Encyclopedia of Genes and Genomes (KEGG). We displayed the top 15 significantly enriched GO pathway terms, containing biological process (BP), cellular component (CC), and molecular function (MF), and the top 15 significantly enriched KEGG pathway terms. Several pathways are related to neurons, such as dense core granule localization, neuron cellular homeostasis, neuron projection cytoplasm, and synaptic vesicle cycle (Figure S1A). Importantly, the 225 differentially expressed proteins, including 96 downregulated and 129 upregulated proteins, were displayed by a volcano plot (Figure 1D) and heatmap (Figure 1E). These differential proteins contained two TRIM family proteins, TRIM2 and TRIM32. Given that TRIM32 has been reported in PD [11], we focus on studying the role of TRIM2 in PD. In addition, TRIM2 expression is declined in the substantia nigra tissues from postmortem human brains of PD patients (*n* = 16) compared to the normal tissues (*n* = 9) through mining data from the GSE7621 dataset (Figure 1F, *p* < 0.05). We verified the decrease of TRIM2 protein in the 6‐OHDA‐induced rat SNc by western blot (Figure 1G, *p* < 0.01). It was suggested that TRIM2 might play a key role in the 6‐OHDA‐lesioned PD rat model.

### 3.2 TRIM2 Overexpression Relieved Neurobehavioral Deficits and Improved Dopaminergic Neuron Loss of the SNc in 6‐OHDA‐Lesioned PD Rats

**FIGURE 2:**
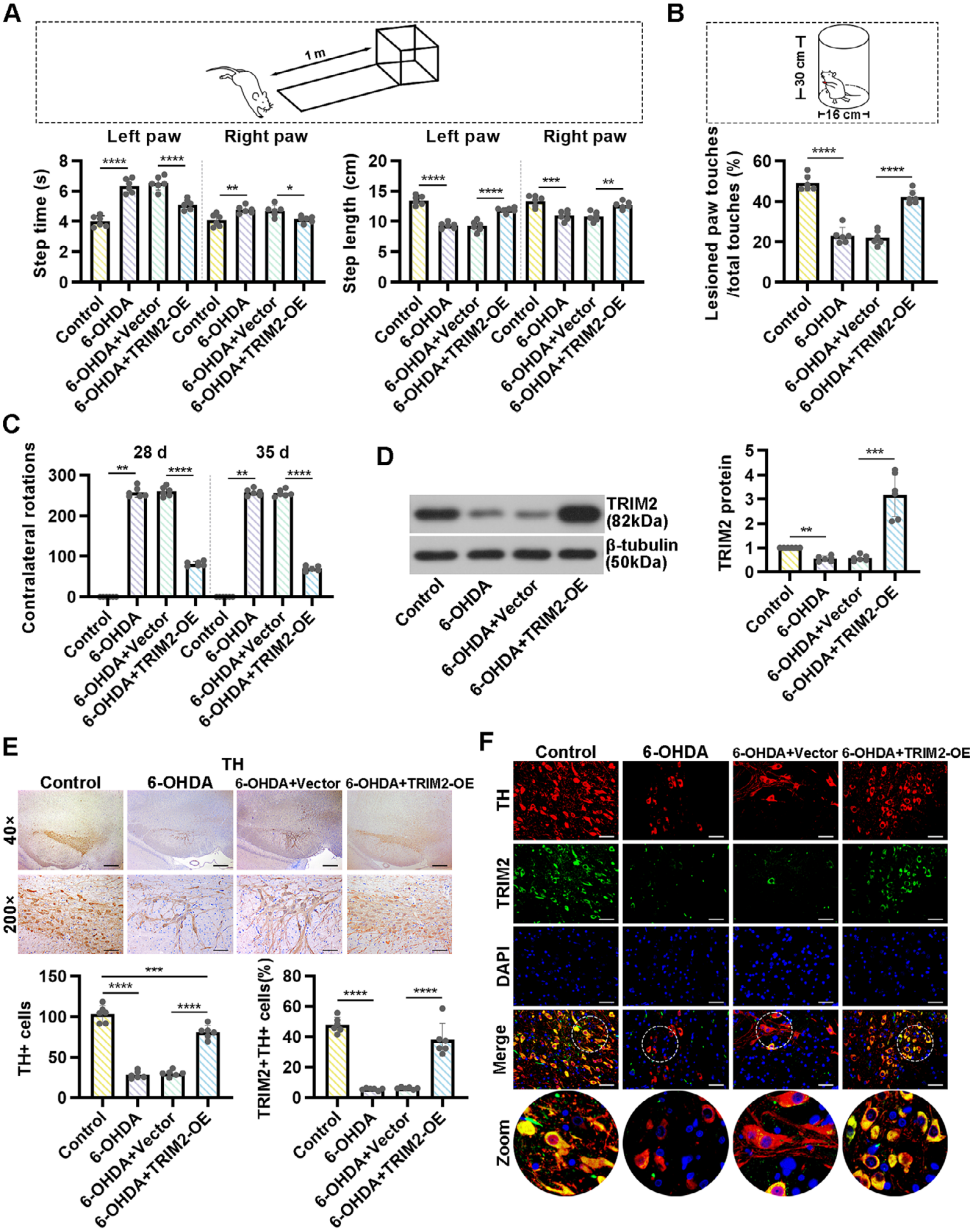
TRIM2 overexpression ameliorated the behavioral deficits and improved dopaminergic neuron loss in 6‐OHDA‐induced PD rats. Four weeks prior to the 6‐OHDA lesion, the rats received an injection of an adeno‐associated virus type 2 (AAV2) vector to overexpress TRIM2. (A) The stepping test was performed on day 28 after the 6‐OHDA injection. The time taken for the rat to complete a 1‐m distance using each paw (step time) and the total distance of 1 m divided by the number of steps made by each paw (average step length) were calculated. The right paw was tested first, followed by the left paw. (B) Cylinder test was conducted on day 28 post‐6‐OHDA injection. The number of touches made by each paw was recorded within 3 min. The percentage of touches made by the lesioned paw relative to the total touches was presented. (C) Apomorphine‐stimulated contralateral rotations were assessed on days 28 and 35 following 6‐OHDA injection. (D) TRIM2 protein levels were analyzed in the rat SNc by western blot. (E) Representative IHC staining for TH in the rat SNc. 40×, scale bar = 500 μm; 200×, scale bar = 100 μm. (F) Immunofluorescence (IF) double‐labeling technique displayed the expression of TRIM2 (green) and TH (red) in the SNc. Nuclei were counterstained with DAPI (blue). 400×, scale bar = 50 μm. *n* = 6 per group. *p < 0.05, **p < 0.01, ***p < 0.001, ****p < 0.0001.

Four weeks after the 6‐OHDA injection, the stepping test was performed. The 6‐OHDA‐treated rats required a longer time to run up a 1‐m slope and return to the cage, which was presented in both the left and right paws. In addition, 6‐OHDA reduced the step length of the left and right paw. However, these effects were counteracted by AAV2 vector‐mediated overexpression of TRIM2 in the PD rats. Moreover, the effects were more pronounced in the left paw (lesioned paw) than the right paw (Figure 2A, *p* < 0.05). The results of the cylinder test revealed that 6‐OHDA rats made fewer lesioned paw touches compared to the control rats. In contrast, overexpressing TRIM2 relieved the inhibitory effect of 6‐OHDA on lesioned paw touches (Figure 2B, *p* < 0.0001). Additionally, 6‐OHDA stimulated apomorphine‐induced contralateral rotation, which was diminished by TRIM2 on days 28 and 35 following 6‐OHDA injection (Figure 2C, *p* < 0.01). After behavioral testing in the rats, the SNc were collected for the following experiments. TRIM2 protein expression was increased by AAV2‐mediated TRIM2 overexpressing vector treatment in the 6‐OHDA‐injected rat SNc (Figure 2D, *p* < 0.01). Furthermore, 6‐OHDA‐reduced TH‐positive neurons were restored by TRIM2 overexpression (Figure 2E, *p* < 0.0001). Notably, the TH‐positive neurons in the 6‐OHDA+TRIM2‐OE group remained lower than those in the control group (*p* < 0.001), indicating that TRIM2 overexpression afforded partial but not complete neuroprotection against 6‐OHDA‐induced dopaminergic neuron loss. IF double staining revealed that expression and co‐localization of TH and TRIM2 were decreased by 6‐OHDA, while TRIM2 overexpression reversed the phenomenon (Figure 2F, *p* < 0.0001). These results demonstrated that TRIM2 was capable of alleviating neurobehavioral dysfunction and ameliorating the injury of dopaminergic neurons induced by 6‐OHDA in the SNc of 6‐OHDA‐lesioned PD rats.

### 3.3 TRIM2 Inhibited Oxidative Stress Response and Dopamine Neuron Apoptosis of the SNc in 6‐OHDA‐Treated PD Rats

**FIGURE 3:**
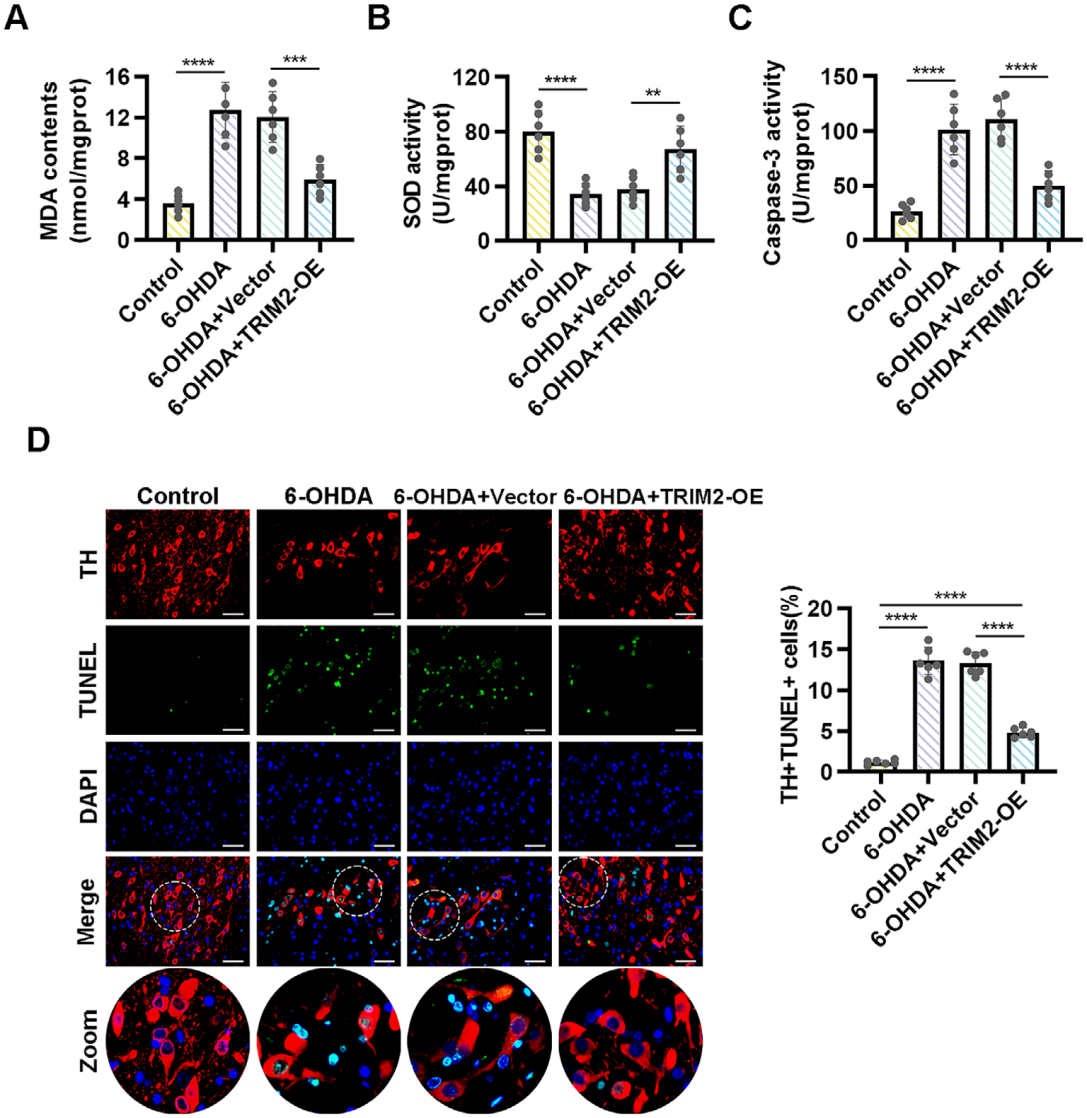
Overexpressing TRIM2 attenuated 6‐OHDA‐induced oxidative stress and apoptosis in the rat SNc. (A) Measurement of malondialdehyde (MDA) contents in the SNc. (B) Assessment of superoxide dismutase (SOD) activity in the SNc. (C) Caspase‐3 activity detection in the SNc. (D) Representative staining images of TUNEL (green) and TH (red) in the SNc. Nuclei were counterstained with DAPI (blue). 400×, scale bar = 50 μm. *n* = 6 per group. **p < 0.01, ***p < 0.001, ****p < 0.0001.

To investigate the protective effects of TRIM2 against 6‐OHDA‐induced neurotoxicity, we evaluated key markers of oxidative stress and apoptosis in the rat SNc. As depicted in Figure 3A,B, overexpression of TRIM2 attenuated 6‐OHDA‐induced oxidative damage, as evidenced by a reduction in MDA levels (*p* < 0.001) and an increase in SOD activity (*p* < 0.01) in the PD rat SNc. Moreover, TRIM2 overexpression effectively suppressed apoptosis, indicated by a decrease in caspase‐3 activity in the PD rat SNc (Figure 3C, *p* < 0.0001). TUNEL and TH staining revealed that TH and TUNEL‐positive cells were enhanced by 6‐OHDA, while TRIM2 suppressed the number of these cells in the SNc (Figure 3D, *p* < 0.0001). Similarly, the cells in the 6‐OHDA+TRIM2‐OE group remained higher than those in the control group (*p* < 0.0001), further supporting that TRIM2 overexpression partially attenuated 6‐OHDA‐induced apoptosis in dopaminergic neurons. Collectively, these results uncovered that TRIM2 overexpression alleviated 6‐OHDA‐induced oxidative stress and dopamine neuron apoptosis in the rat SNc.

### 3.4 TRIM2 Repressed 6‐OHDA‐Stimulated Cell Injury, Apoptosis and Oxidative Stress in the Differentiated BE (2)‐M17 Cells

**FIGURE 4:**
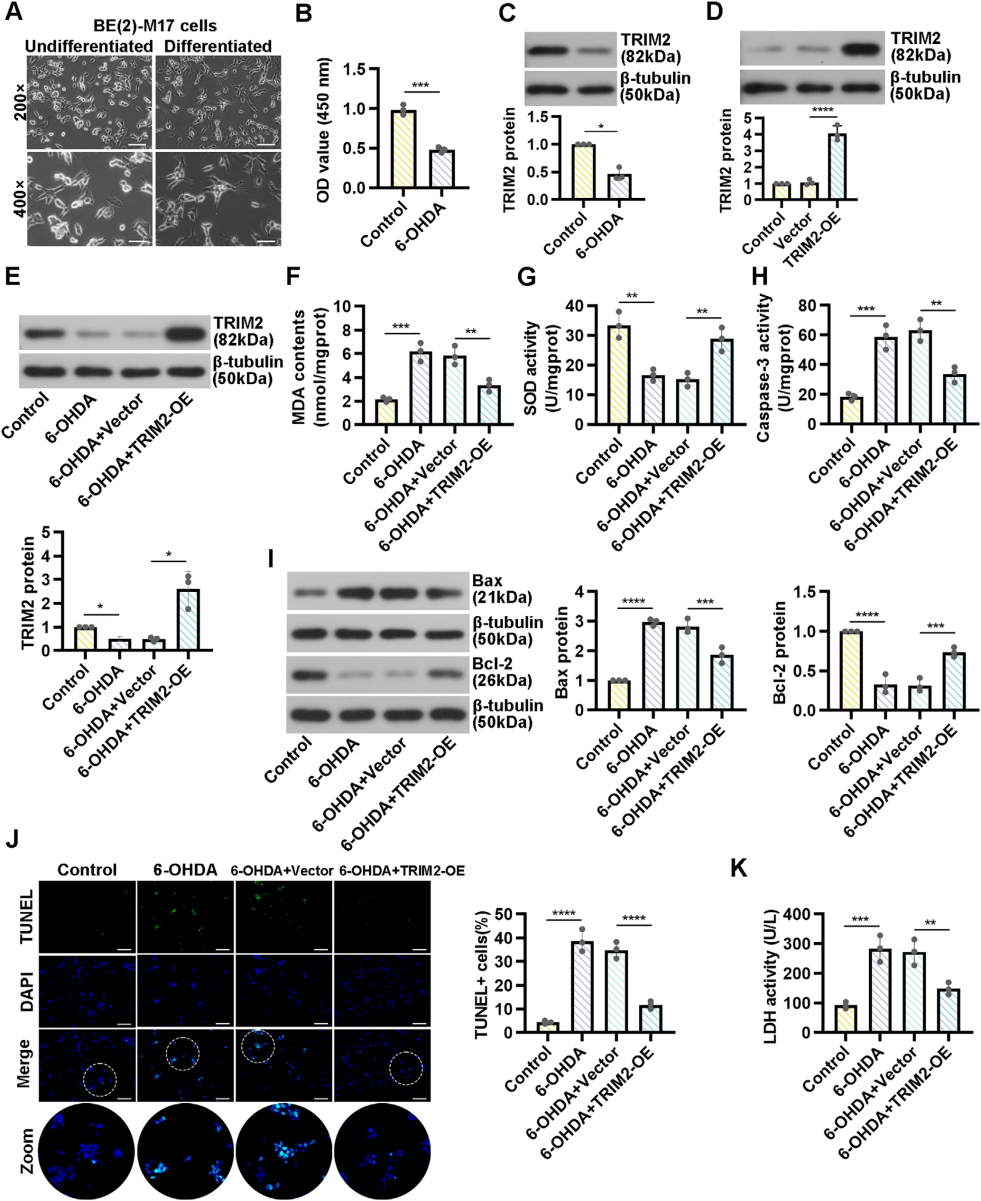
Overexpression of TRIM2 alleviated 6‐OHDA‐induced cell apoptosis and oxidative stress in differentiated BE (2)‐M17 cells. (A) Morphological changes of BE (2)‐M17 cells following differentiation induced by trans‐retinoic acid (RA). 200×, scale bar = 100 μm; 400×, scale bar, 50 μm. (B) Cell viability was assessed by CCK‐8 assay in the differentiated BE (2)‐M17 cells after 6‐OHDA treatment. (C) Western blot analysis of TRIM2 protein expression in the cells. (D) Validation of TRIM2 expression by western blot in the cells transfected with the TRIM2‐overexpressing plasmid (TRIM2‐OE) or empty vector (Vector). (E) TRIM2 protein levels in the transfected cells challenged with 6‐OHDA were evaluated by western blot. (F) MDA contents in the cells. (G) SOD activity in the cells. (H) Caspase‐3 activity determination in the cells. (I) The protein level measurement of the pro‐apoptotic factor Bax and the anti‐apoptotic factor Bcl‐2. (J) TUNEL staining to detect apoptotic cells (green). Nuclei were counterstained with DAPI (blue). 200×, scale bar = 100 μm. (K) Lactate dehydrogenase (LDH) activity detection in the cells. *n* = 3 per group. *p < 0.05, **p < 0.01, ***p < 0.001, ****p < 0.0001.

BE (2)‐M17 cells were treated with RA to induce neuronal differentiation. As shown in Figure 4A, the differentiated cells displayed a neuron‐like triangular shape with more apparent synapses. The differentiated cells were used for subsequent experiments. Cell viability was reduced after 6‐OHDA treatment (Figure 4B, *p* < 0.001). 6‐OHDA inhibited TRIM2 protein expression in the cells (Figure 4C, *p* < 0.05). Overexpressed TRIM2 vector‐transfected cells exhibited more protein contents of TRIM2 compared with control plasmid‐transfected cells (Figure 4D, *p* < 0.0001). Furthermore, TRIM2 overexpression was efficiently achieved in the transfected cells subjected to 6‐OHDA (Figure 4E, *p* < 0.05). 6‐OHDA enhanced MDA contents in the differentiated BE (2)‐M17 cells, while TRIM2 reduced the contents (Figure 4F, *p* < 0.01). Additionally, SOD activity suppressed by 6‐OHDA was restored by TRIM2 overexpression in the cells (Figure 4G, *p* < 0.01). The anti‐apoptotic effect of TRIM2 was substantiated by a marked decrease in caspase‐3 activity (Figure 4H, *p* < 0.01) and pro‐apoptotic Bax expression (Figure 4I, *p* < 0.001), as well as an increase in anti‐apoptotic Bcl‐2 expression (Figure 4I, *p* < 0.001). Besides, TRIM2 overexpression suppressed 6‐OHDA‐stimulated TUNEL‐positive cells (Figure 4J, *p* < 0.0001). Moreover, TRIM2 overexpression reduced LDH release in 6‐OHDA‐challenged cells (Figure 4K, *p* < 0.01). These results suggested that TRIM2 conferred protection against 6‐OHDA‐induced cell injury, apoptosis, and oxidative stress in an in vitro neuron model.

### 3.5 The Interaction Between TRIM2 and ELAVL1 in Vivo and in Vitro

**FIGURE 5:**
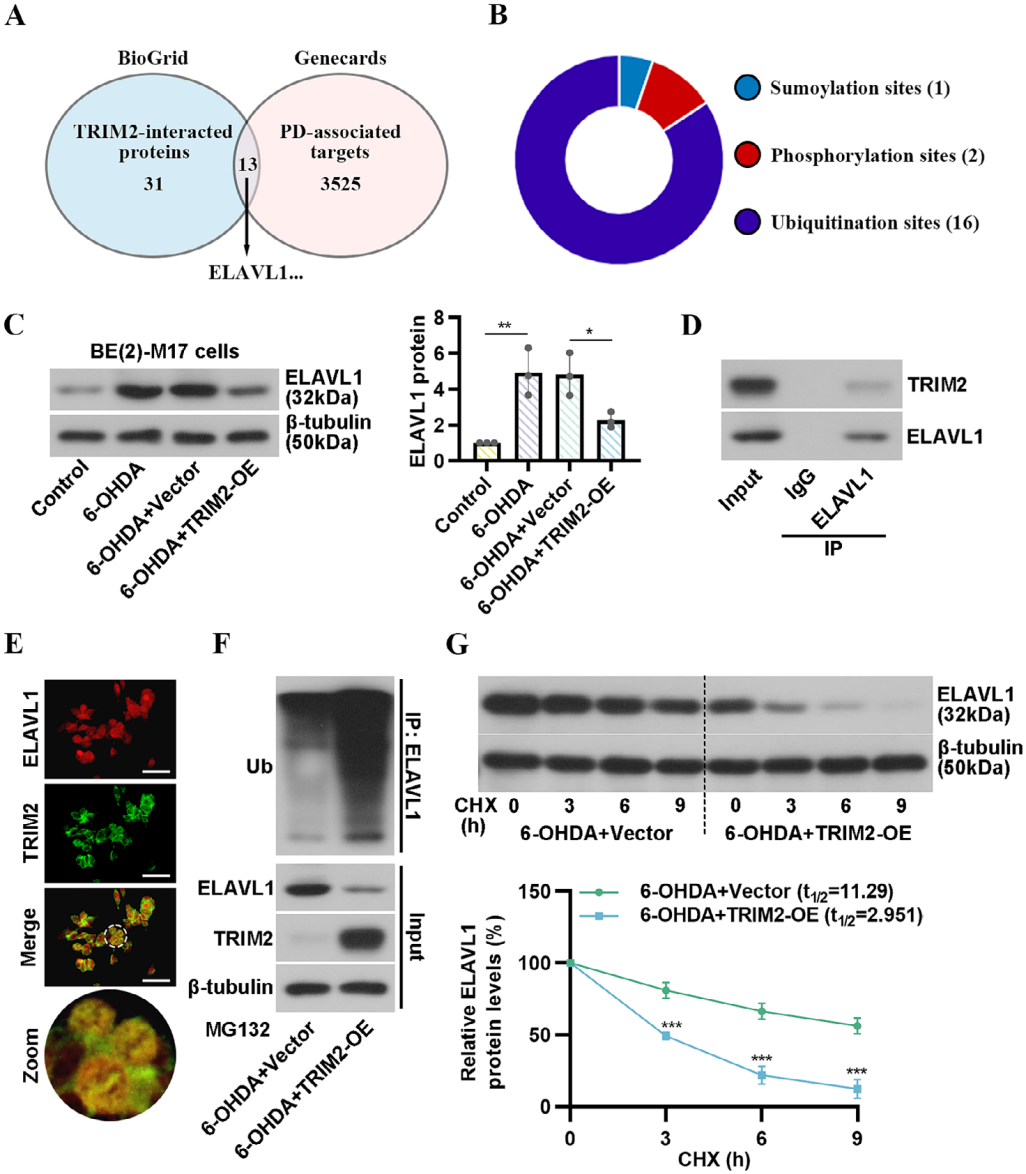
TRIM2 was capable of interacting with ELAVL1 and promoting the ubiquitination and degradation of ELAVL1. (A) Identification of ELAVL1 as a potential TRIM2‐interacting protein relevant to PD. A Venn diagram shows the overlap between 44 protein interactors of human TRIM2 from the BioGRID database (screening criteria: High‐throughput) and 3538 targets associated with PD from Genecards (relevance score > 10), yielding 13 candidates, including ELAVL1. (B) The presentation of ubiquitination sites of the ELAVL1 protein via retrieving the BioGRID database. (C) Western blot analysis of ELAVL1 protein levels in the BE (2)‐M17 cells. (D) Interaction between TRIM2 and ELAVL1 was assessed by co‐immunoprecipitation (Co‐IP) in non‐transfected cells. (E) Co‐localization of TRIM2 (green) and ELAVL1 (red) was visualized by IF staining in non‐transfected cells. 400×, scale bar = 50 μm. (F) The cells transfected with TRIM2‐OE or vector were treated with 6‐OHDA, followed by exposure to the proteasome inhibitor MG132. ELAVL1 ubiquitination was detected in the cells. (G) The cells were treated with cycloheximide (CHX) for the indicated times. Quantification of ELAVL1 protein levels revealed that TRIM2 accelerated ELAVL1 degradation. *n* = 3 per group. *p < 0.05, **p < 0.01, ***p < 0.001.

ELAVL1 was considered a promising candidate, residing at the intersection of 44 TRIM2 interactors from BioGRID (screening criteria: High‐throughput) and 3538 high‐relevance PD‐associated targets from Genecards (relevance score > 10) (Figure 5A). It was presented that the ELAVL1 protein might possess multiple potential ubiquitination sites on the basis of the BioGRID database (Figure 5B). ELAVL1 protein levels were diminished by TRIM2 overexpression in the differentiated BE (2)‐M17 cells following 6‐OHDA intervention (Figure 5C, *p* < 0.05). Notably, the binding between TRIM2 and ELAVL1 was confirmed by Co‐IP (Figure 5D), which was further supported by the co‐localization of TRIM2 and ELAVL1 as revealed by IF staining in the differentiated BE (2)‐M17 cells (Figure 5E). Crucially, in the presence of the proteasome inhibitor MG132, TRIM2 overexpression enhanced the ubiquitination of ELAVL1 in the 6‐OHDA‐treated cells (Figure 5F). CHX chase assay was performed to further assess the impact of TRIM2 overexpression on the stability of the ELAVL1 protein in the cells. Western blot analysis of ELAVL1 expression at various time points after CHX addition unveiled a rapid decline in ELAVL1 protein levels. Subsequent quantification determined that the half‐life of ELAVL1 declined from 11.29 h in the empty vector‐transfected 6‐OHDA cells to 2.951 h in the overexpressed TRIM2 vector‐transfected 6‐OHDA cells, suggesting that TRIM2 overexpression expedited the degradation of ELAVL1 protein (Figure 5G, *p* < 0.001). The results demonstrated that TRIM2 directly interacted with ELAVL1 and promoted its ubiquitin‐mediated proteasomal degradation in the 6‐OHDA‐treated differentiated BE (2)‐M17 cells.

In vivo, the upregulation of ELAVL1 mRNA induced by 6‐OHDA was reversed by TRIM2 in the PD rat SNc (Figure S2A, *p* < 0.0001). Western blot analysis confirmed similar changes at the protein levels (Figure S2B, *p* < 0.01). IF double staining for ELAVL1 with TRIM2 showed that ELAVL1 was co‐localized with TRIM2 in the SNc (Figure S2C). Additionally, TRIM2 and ELAVL1 expression was observed in TH^+^ dopaminergic neurons, GFAP^+^ astrocytes, and Iba1^+^ microglia (Figures 2F, S2D, and S3A,B). The primary focus of our study is their role in dopaminergic neurons. Interestingly, an upregulation of ELAVL1 levels was shown in the substantia nigra tissues from PD patients compared to controls based on the GSE7621 dataset (Figure S4A, *p* < 0.01). Furthermore, Pearson correlation analysis between TRIM2 and ELAVL1 expression values across all samples displayed a negative correlation (r = −0.4244, *p* < 0.05; Figure S4B), supporting the negative regulatory relationship identified in our mechanistic studies. Overall, it was confirmed that there was a close connection between TRIM2 and ELAVL1 in vitro and in vivo.

### 3.6 TRIM2 Mediated Neuroprotection Against 6‐OHDA‐Induced Oxidative Stress and Apoptosis by Facilitating ELAVL1 Degradation

**FIGURE 6:**
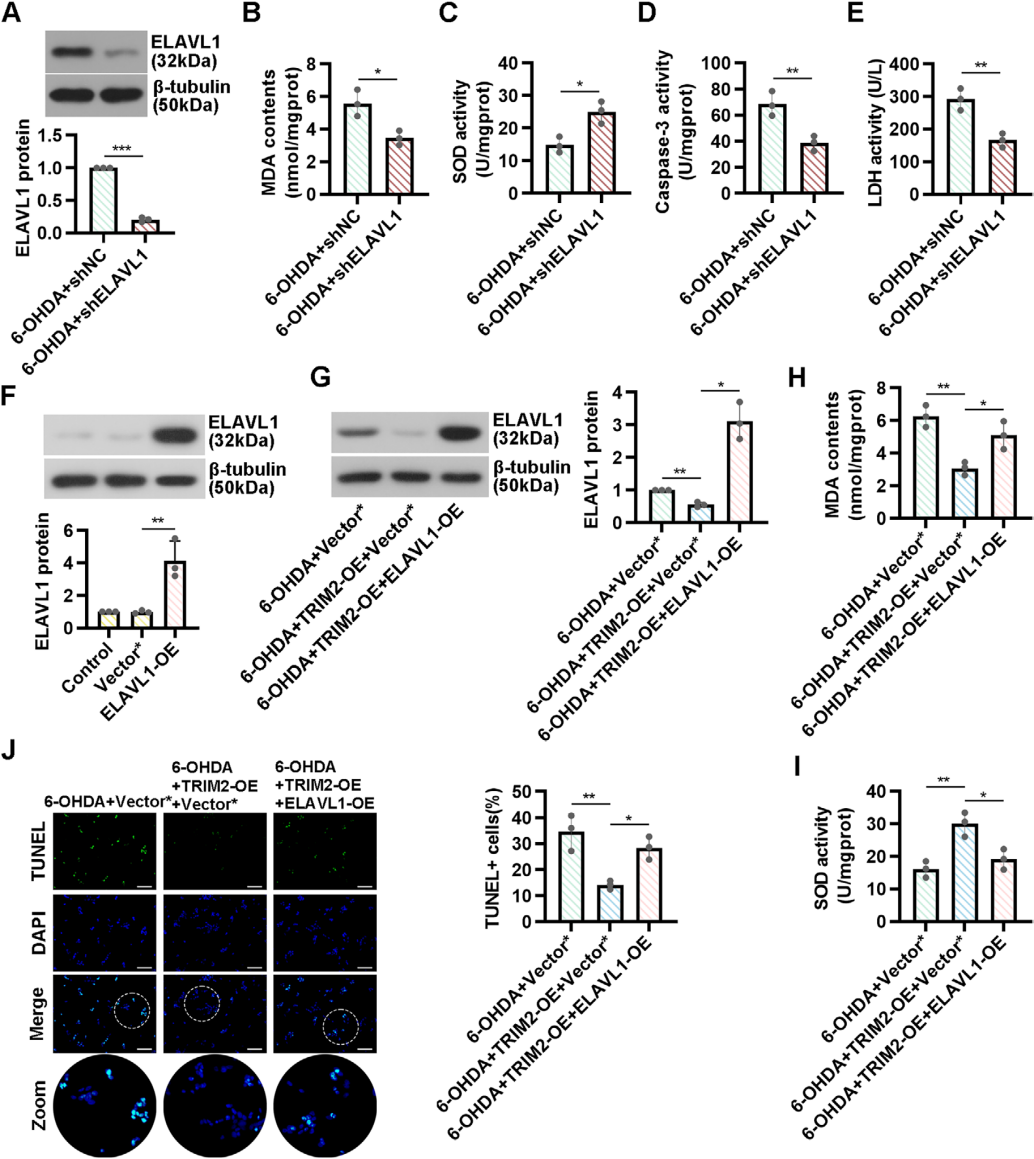
ELAVL1 was required for the protective effects of TRIM2 against 6‐OHDA‐induced oxidative stress and apoptosis in the BE (2)‐M17 cells. (A) ELAVL1 protein levels in the BE(2)‐M17 cells transfected with targeted‐ELAVL1 shRNA (shELAVL1) or negative control (shNC), followed by 6‐OHDA treatment. (B) MDA levels. (C) SOD activity. (D) Caspase‐3 activity. (E) LDH activity in the cells. (F) ELAVL1 protein levels were validated by western blot in the cells transfected with an ELAVL1‐overexpressing (ELAVL1‐OE) plasmid or the corresponding empty vector (Vector*). (G) ELAVL1 expression was measured in the cells transfected with TRIM2‐OE and ELAVL1‐OE under 6‐OHDA treatment. (H) MDA contents. (I) SOD activity in the cells. (J) Cell apoptosis was evaluated by TUNEL staining (green). Nuclei were counterstained with DAPI (blue). 200×, scale bar = 100 μm. *n* = 3 per group. *p < 0.05, **p < 0.01, ***p < 0.001.

Western blot analysis confirmed that shELAVL1 transfection reduced ELAVL1 protein levels compared to shNC in the differentiated BE(2)‐M17 cells under 6‐OHDA treatment (Figure 6A, *p* < 0.001). ELAVL1 knockdown attenuated MDA levels (Figure 6B, *p* < 0.05). Meanwhile, SOD activity was restored upon shELAVL1 transfection (Figure 6C, *p* < 0.05). In addition, ELAVL1 knockdown induced a decrease in caspase‐3 activity (Figure 6D, *p* < 0.01) and suppressed LDH activity (Figure 6E, *p* < 0.01). These results indicated that ELAVL1 downregulation exhibited a protective role in the 6‐OHDA‐treated cells. Importantly, the successful overexpression of ELAVL1 was first confirmed in the differentiated BE (2)‐M17 cells by western blot (Figure 6F, *p* < 0.01). The cells were transfected with ELAVL1 or TRIM2 overexpressing vectors and treated with 6‐OHDA. The suppressive effect of TRIM2 overexpression on ELAVL1 protein levels was reversed by concurrent ELAVL1 overexpression (Figure 6G, *p* < 0.05). TRIM2 overexpression weakened oxidative stress, evidenced by reduced MDA contents and enhanced SOD activity, which was compromised by ELAVL1 overexpression (Figure 6H,I, *p* < 0.05). The anti‐apoptotic effect conferred by overexpressing TRIM2, as assessed by TUNEL staining, was attenuated upon ELAVL1 overexpression in the cells (Figure 6J, *p* < 0.05). In summary, the experimental data proved that ELAVL1 was required for the protective role of TRIM2 against 6‐OHDA‐induced oxidative stress and apoptosis in the differentiated BE (2)‐M17 cells.

## 4 Discussion

In the present study, proteomic sequencing revealed downregulation of TRIM2 in a 6‐OHDA‐induced rat PD model, which was further confirmed by analysis of the GSE7621 microarray dataset. We speculated that TRIM2 might be involved in the pathogenesis of PD. In vivo, TRIM2 ameliorated behavioral deficits and attenuated oxidative stress and apoptosis in the SNc of 6‐OHDA‐lesioned rats. In vitro, TRIM2 overexpression suppressed 6‐OHDA‐caused oxidative stress and apoptosis in the BE (2)‐M17 cell model of PD. Importantly, our results elucidated that TRIM2‐mediated neuroprotection was mechanistically linked to the ubiquitin‐dependent degradation of ELAVL1. These findings might suggest a promising novel neuroprotective strategy for PD.

A number of TRIM family proteins have been reported to play pivotal roles in PD. For instance, TRIM11 has been shown to mitigate the loss of dopaminergic neurons in the substantia nigra of PD mice and alleviate α‐synuclein (α‐Syn)‐mediated motor deficits [28]. Knockout of TRIM27 suppresses apoptosis and loss of dopaminergic neurons in PD mouse models [29]. TRIM28 stabilizes and promotes the nuclear accumulation of both α‐Syn and microtubule‐associated protein tau, thereby accelerating PD progression [30]. Furthermore, a study indicates that silencing TRIM17 reduces α‐Syn expression, whereas knockout of TRIM41 increases it, both being implicated in PD pathogenesis [31]. These findings collectively highlight the neuroprotective or detrimental roles of TRIM family proteins in PD. In our study, TRIM2 was identified as exerting a neuroprotective function in the 6‐OHDA‐induced PD rat model. We first established a 6‐OHDA‐induced PD model, as described in previous studies [32, 33], and conducted proteomic sequencing to explore the differentially expressed proteins in the SNc tissues from PD rats compared to healthy rats. Interestingly, two TRIM family members, TRIM2 and TRIM32, were found to be downregulated in the SNc of PD rats. Literature has revealed that TRIM32 promotes neuronal apoptosis by reducing XIAP levels in cellular PD models [11]. In contrast, the role of TRIM2 in PD had not been previously reported. TRIM2 can ubiquitinate Bim and exerts neuroprotection in rapid ischemic tolerance [17]. Moreover, TRIM2 interacts with ALG‐2 interacting protein X and plays a critical role in early neural development [12]. Herein, we found that TRIM2 inhibited neuronal damage in PD in vivo and in vitro models.

Neuronal oxidative stress and apoptosis are key contributors to the initiation and progression of PD [34, 35]. In recent years, multiple researchers have focused on identifying molecules or compounds that target neuronal oxidative stress and apoptosis. Paraoxonase 2 confers neuroprotection by attenuating neuronal oxidative stress [36]. Agmatine exerts neuroprotective effects in rotenone‐induced PD rats by reducing oxidative stress markers such as MDA levels and enhancing the release of the antioxidant enzyme SOD [37]. Additionally, Parthanatos serves as a cell apoptosis inhibitor, targeting Parthanatos‐associated apoptosis‐inducing factor nuclease, and has been shown to prevent neuronal damage in PD mice [38]. Importantly, TRIM2 is capable of activating ROS‐related NRF2/ARE signaling [19], which counteracts oxidative stress response and is a neuroprotective pathway in PD [20, 21]. In addition, TRIM2 protects from retinal ganglion cell apoptosis [18]. Consistent with previous research, TRIM2 resisted PD by inhibiting neuronal oxidative stress and apoptosis. It is noteworthy that TRIM2 overexpression only partially rescued TH‐positive neuron loss as well as reduced the number of TH and TUNEL double‐positive cells. This partial protection is likely attributable to the fact that 6‐OHDA triggers multiple concurrent cell death pathways beyond apoptosis, such as necroptosis [39, 40], ferroptosis [41, 42], and pyroptosis [43, 44], which may not be controlled by TRIM2.

Based on the BioGRID database, ELAVL1 was identified as a putative interactor of TRIM2. ELAVL1 has been implicated in the pathogenesis of various human diseases, including cancers [36, 45, 46], pulmonary fibrosis [47], osteoporosis [48], traumatic brain injury [49], and PD [50]. Notably, targeted suppression of ELAVL1 expression by miR‐9a‐5p has been shown to mitigate the pathology of traumatic brain injury [49]. Suppression of ELAVL1 stimulates the differentiation of neural stem cells into neurons, contributing to improved motor function, reduced neural damage, and enhanced spinal cord repair [51]. Furthermore, knockdown of ELAVL1 has been shown to inhibit the NACHT, LRR, and PYD domains‐containing protein 3 (NLRP3)‐mediated pyroptosis activation and mitigate neuronal injury in PD [50]. ELAVL1 inhibition ameliorates oxidative stress and apoptosis, thereby alleviating neuronal damage in Alzheimer's disease [52]. In a word, targeting ELAVL1 confers neuroprotection against neuronal injury. Our results elucidated that TRIM2 protected dopaminergic neurons from 6‐OHDA‐induced oxidative stress and apoptosis via promoting the ubiquitination‐mediated degradation of ELAVL1.

Several limitations of this study should be acknowledged. First, only male SD rats were used in our in vivo experiments. PD exhibits well‐recognized sex differences in epidemiology, clinical phenotype, and disease progression [53]. Whether the neuroprotective role of TRIM2 observed in male rats holds true in females remains to be investigated. Second, we employed only the 6‐OHDA‐induced PD model. While 6‐OHDA is a well‐established model for studying dopaminergic neuron degeneration, it does not recapitulate all disease characteristics in PD. Thus, it is necessary to use other PD models, such as α‐synuclein preformed fibrils injection [54], for complementary validation. Third, our study mainly focused on dopaminergic neurons, while TRIM2 and ELAVL1 were also detected in astrocytes and microglia. Astrocytes and microglia are known to exacerbate dopaminergic neuron loss by releasing pro‐inflammatory cytokines and reactive oxygen species in 6‐OHDA‐triggered PD models [55, 56]. The TRIM2‐ELAVL1 axis may also contribute to neuroprotection through modulation of the glial microenvironment. The precise contribution of glial TRIM2‐ELAVL1 signaling to neuroprotection remains to be elucidated. Fourth, we did not detect the effects of ELAVL1 overexpression in vivo. Future studies using AAV‐mediated ELAVL1 overexpression in the rat SNc are needed to further establish its pathogenic role in PD.

## 5 Conclusion

In summary, our study established TRIM2 as a critical regulator in PD models, alleviating neurodegeneration and oxidative stress. The neuroprotective roles were supported by its targeted ubiquitination and degradation of ELAVL1. The study confirmed that the TRIM2‐ELAVL1 axis served as a pivotal mechanism in PD pathogenesis, unlocking a new avenue for PD therapy.

## Author Contributions

W.L. performed experiments, performed data analysis, prepared figures, and wrote the original draft. W.S. assisted with data analysis and figure preparation. Z.Z. assisted with data analysis. K.S. performed manuscript revisions. J.J. designed the study, performed manuscript revisions, and supervised the research. All authors have read and approved the final version of the manuscript.

## Funding

This research was funded by the Medical Science and Technology Research Program Jointly Established Project of Henan Province (Grant No. LHGJ20230163).

## Ethics Statement

The present study was approved by the First Affiliated Hospital of Zhengzhou University (2025‐KY‐0297).

## Conflicts of Interest

The authors declare no conflicts of interest.

## Supporting information


**Figure S1:** Gene Ontology (GO) and Kyoto Encyclopedia of Genes and Genomes (KEGG) pathway enrichment analysis of the differentially expressed proteins (Fold Change > 1.5 or < 0.67, *p* < 0.05) on the basis of proteomic sequencing. (A) The top 15 significantly enriched GO pathway terms, containing biological process (BP), cellular component (CC), and molecular function (MF), as well as the top 15 significantly enriched KEGG pathway terms, were presented. The pathways related to neurons were highlighted in red.
**Figure S2:** TRIM2 reduced ELAVL1 expression in the PD rat SNc. (A) Real‐time PCR analysis of ELAVL1 mRNA levels in the rat SNc. (B) ELAVL1 protein expression in the SNc. (C) Double IF staining for TRIM2 (red) and ELAVL1 (green) in the SNc. Nuclei were counterstained with DAPI (blue). 400×, scale bar = 50 μm. (D) Double IF staining for TH (red) and ELAVL1 (green) in the SNc. Nuclei were counterstained with DAPI (blue). 400×, scale bar = 50 μm. *n* = 6 per group. **p < 0.01, ****p < 0.0001.
**Figure S3:** TRIM2 or ELAVL1 expression in astrocytes and microglia of the rat SNc. (A) Double IF staining for TRIM2 (green) together with GFAP (astrocytes, red) or Iba1 (microglia, red) in the SNc. Nuclei were counterstained with DAPI (blue). 400×, scale bar = 50 μm. (B) Double IF staining for ELAVL1 (green) with GFAP (red) or Iba1 (red) in the SNc. Nuclei were counterstained with DAPI (blue). 400×, scale bar = 50 μm.
**Figure S4:** Expression of ELAVL1 and correlation between ELAVL1 and TRIM2 expression in human PD substantia nigra based on the GSE7621 dataset. (A) Upregulated ELAVL1 expression in the substantia nigra tissues from 16 postmortem human brains of PD patients compared to 9 healthy controls. (B) Pearson correlation analysis between TRIM2 and ELAVL1 expression values in the dataset (r = −0.4244, p < 0.05). **p < 0.01.

## Data Availability

The data that support the findings of this study are available from the corresponding author upon reasonable request.
