# Supplementary material for: Neuroprotective Role of E3 Ubiquitin Ligase TRIM2 in Parkinson's Disease: Attenuation of Oxidative Stress and Apoptosis via Promoting ELAVL1 Ubiquitination
Source: CNS Neurosci Ther. 2026 Aug 12;32(8):e71075. doi: 10.1002/cns.71075 (PMC13465207; doi:10.1002/cns.71075)
Supplement: Supplementary file 1 [file CNS-32-e71075-s001.docx]

**Supporting information**

**Materials and methods**

**Animals**

Ten-week age male Sprague-Dawley (SD) rats weighing approximately 250 g were purchased from Changsheng Biotechnology Co., Ltd. The PD rat model was established with reference to previous literatures [1-3]. The SD rats were intraperitoneally injected with 20 mg/kg imipramine (I863188, Macklin, Shanghai, China) [4]. The rats were anesthetized and fixed in a stereotaxic apparatus 30 min later. Then, 14 μg 6-OHDA (H873296, Macklin, Shanghai, China) was dissolved in 4 μL 0.02% ascorbic acid (3.5 µg/µL, A800295, Macklin, Shanghai, China). The solution was delivered slowly into the rat medial forebrain bundles (-4.4 mm anteroposterior, -1.2 mm mediolateral, and -7.8 mm dorsoventral relative to the bregma) on the right at an infusion rate of 1 µL/min, using a 26-gauge needle attached to a Hamilton syringe. The control rats received an equivalent volume of 0.02% ascorbic acid vehicle following the same procedure. The rat rotational behaviors were assessed on the 7th day after 6-OHDA injection. The rats were administered 0.5 mg/kg apomorphine (A276058, Aladdin, Shanghai, China) subcutaneously in the neck region. The number of contralateral rotations was recorded 3 min after apomorphine injection. The rats exhibiting more than 100 turns within 30 min were selected for subsequent experiments. Besides, we also recorded the rat contralateral rotations on the 28th and 35th days after 6-OHDA injection. After the completion of the rotational behavior test on the 35th day after 6-OHDA injection, the rats were euthanized. The SNc tissues on the injured side were isolated for subsequent experiments.

**Label-free proteomics**

The SNc tissues of control and 6-OHDA-treated rats were ground into powder using liquid nitrogen and centrifuged to extract the supernatant. Determination of protein concentration using the Bradford method. The qualified samples were entrusted to Beijing Qinglian Biotechnology Co., Ltd. for label-free proteomics sequencing (n=3). The differentially expressed proteins were identified based on the following criteria: Fold Change > 1.5 for upregulation or Fold Change < 0.67 for downregulation, and a *p* value < 0.05.

**Behavioral observations**

In addition to rotational behavior testing, we also conducted stepping experiments and cylinder experiments on rats using methods from previous literature [5,6]. The stepping test was conducted using a 1-meter-long wooden ramp, which was connected to the rat’s home cage. Prior to testing, the rats were acclimatized to the experimenter’s handling through daily contact for 3 consecutive days. Over the next 1-2 days, the rats were trained to voluntarily run up the ramp to return to their home cage. The experimenter gently restrained the rat’s hindlimbs with one hand, slightly lifting its posterior off the ground. The forelimb not being assessed was gently immobilized by the other hand. The rat was then placed on the ramp, and the time taken for it to traverse the 1-meter distance using only the tested forelimb was recorded as the stepping time. The average stride length for the tested forelimb was calculated by dividing the 1-meter distance by the number of steps taken. The right forepaw was tested first, followed by the left forepaw for each animal.

The cylinder test was employed to assess spontaneous forelimb use. Each rat was placed individually into a transparent plexiglass cylinder (16 cm in diameter, 30 cm in height) and allowed to explore freely for a 3-minute session. The number of independent contacts made by each forelimb against the wall was recorded during exploration. The percentage of touches made by the impaired paw to the total number of touches was calculated.

**Immunohistochemistry (IHC)**

The SNc tissues were fixed and embedded in paraffin. The paraffin-embedded tissues were then sectioned into 5-μm thin slices. Following deparaffinization and rehydration, the sections underwent antigen retrieval. Subsequently, the slices were gently blotted dry with absorbent paper and incubated with 3% H₂O₂ at room temperature for 15 min to quench endogenous peroxidase activity. Next, the sections were blocked with 1% bovine serum albumin (BSA, A602440, Sangon, Shanghai, China) at room temperature for another 15 min. The samples were incubated with tyrosine hydroxylase (TH) polyclonal antibody (1:500, 25859-1-AP, Proteintech, Wuhan, China) overnight at 4℃. Goat anti-rabbit IgG/HRP (SE134, Solarbio, Beijing, China) was applied at room temperature for 45 min. Horseradish catalase DAB color kit (C520017, Sangon, Shanghai, China) was used for color development. The slices were counterstained with hematoxylin (H8070, Solarbio, Beijing, China). TH-positive cells in the SNc were photographed with a microscope (DP73, OLYMPUS, Japan). Subsequently, TH‑positive cells were manually counted using Image‑Pro Plus software in a blinded manner.

**Cell treatment**

Plasmids encoding human TRIM2 (NM_015271) for TRIM2 overexpression (TRIM2-OE), human ELAVL1 (NM_001419) for ELAVL1 overexpression (ELAVL1-OE), or shRNA targeting ELAVL1 for ELAVL1‑knockdown (shELAVL1) and their corresponding controls (Vector or shNC), were transfected into the cells using Lipofectamine 3000 (L3000015, Invitrogen, Thermo Fisher Scientific, USA). The sequence of shELAVL1 is as follows: GGTGAAGTTGAATCTGCAA.

For the determination of the effect of TRIM2 on ELAVL1 degradation, the transfected cells were treated with 80 μM 6-OHDA for 18 h and then treated with 20 μM MG132 (M832899, Macklin, Shanghai, China) for 2 h for subsequent co-immunoprecipitation (Co-IP) assay. Similarly, these cells were treated with 50 μM cycloheximide (CHX, C112766, Aladdin, Shanghai, China) for different times (0, 3, 6, and 9 h) for the detection of ELAVL1 using western blot.

**Cell viability**

The differentiated BE (2)-M17 cells were seeded in a 96-well plate. The cells were placed in a 37℃ and 5% CO_2_ incubator. After treating cells with 80 μM 6-OHDA for 18 h, 10 μL CCK8 was added into each well and incubated in the incubator for 2 h. The OD values were determined at 450 nm using a microplate reader (800TS, BIOTEK, USA).

**Lactate dehydrogenase (LDH) activity**

The lactate dehydrogenase assay kit (A020, Nanjing Jiancheng Bioengineering Institute, Jiangsu, China) was used to determine LDH activity in the cells. After 48 h of cell transfection and treatment with 6-OHDA, the cells were subjected to a centrifugation at 4000 r/min for 5 min and lactate dehydrogenase assay kit (A020, Nanjing Jiancheng Bioengineering Institute, Jiangsu, China) was applied to evaluate LDH activity in the cell supernatant using 0.2 μmol/mL sodium pyruvate standard solution.

**Caspase-3 activity**

Caspase-3 activity in each sample was examined according to the Caspase-3 activity assay kit (BC3830, Solarbio, Beijing, China). The rat SNc tissues were ground and kept on ice for 15 min. Subsequently, the samples were centrifuged at 15000 g for 10 min and maintained on ice for immediate assay. For cell sample preparation, after centrifugation and removal of the supernatant, approximately 10^6^ cells were resuspended and incubated on ice for 15 min, followed by centrifugation at 15000 g for 10 min at 4°C. The supernatants were then collected and kept on ice for subsequent analysis. The protein concentrations in the tissues and cell supernatants were determined using a Bradford Protein Assay Kit (PC0010, Solarbio, Beijing, China). Subsequently, Caspase-3 activity was detected by the kit.

**Immunofluorescence (IF)**

The fixed SNc tissues were embedded using paraffin and cut into 5 μm-thin slices. The slices were subjected to dewaxing and antigen retrieval. Next, the slices were blocked by 1% BSA (A602440, Sangon, Shanghai, China). The samples were incubated with the following primary antibodies overnight at 4℃: TRIM2 mouse antibody (1:200, 67342-1-Ig, Proteintech, Wuhan, China), TH rabbit antibody (1:200, 25859-1-AP, Proteintech, Wuhan, China), GFAP rabbit antibody (1:100, #R22776, Zenbio, Sichuan, China), Iba1 rabbit antibody (1:100, Ab178846, Ancam, UK), or ELAVL1 rabbit antibody (1:100, 11910-1-AP, Proteintech, Wuhan, China). For IF double staining of ELAVL1 and those cellular markers (TH, GFAP and Iba1), ELAVL1 mouse antibody (1:200, 66549-1-Ig, Proteintech, Wuhan, China) was used. Afterwards, the slices were probed using corresponding secondary antibodies, FITC-goat anti-mouse IgG (1:200, SA00003-1, Proteintech, Wuhan, China) or Cy3-goat anti-rabbit IgG (1:200, SA00009-2, Proteintech, Wuhan, China), for 90 min at room temperature. DAPI (D106471, Aladdin, Shanghai, China) was used to cover the tissues for counterstaining nuclei.

For cell IF staining, the cells were fixed using 4% paraformaldehyde (80096618, Sinopharm chemical reagent, Shanghai, China) for 15 min. The cells were penetrated with 0.1% TritonX-100 (ST795, Beyotime, Shanghai, China) for 30 min and covered with 1% BSA (A602440, Sangon, Shanghai, China) for 15 min. The samples were incubated with TRIM2 mouse antibody (1:200, 67342-1-Ig, Proteintech, Wuhan, China) or ELAVL1 rabbit antibody (1:100, 11910-1-AP, Proteintech, Wuhan, China) overnight at 4℃. The secondary antibodies are as follows: FITC-goat anti-mouse IgG (1:200, SA00003-1, Proteintech, Wuhan, China) or Cy3-goat anti-rabbit IgG (1:200, SA00009-2, Proteintech, Wuhan, China). The cells were incubated with the secondary antibodies in the dark for 60 min. DAPI (D106471, Aladdin, Shanghai, China) was used to counterstain nuclei. The images were observed and photographed under a microscope (DP73, OLYMPUS, Japan).

**TUNEL**

In Situ Cell Death Detection Kit (11684795910, Roche, Switzerland) was used for TUNEL detection. For co-detection of TUNEL and IF, 5 μm thick paraffin-embedded sections were deparaffinized, permeabilized, and subjected to antigen retrieval. Subsequently, 50 μL TUNEL reaction mixture, prepared by mixing enzyme solution and label solution at a 1:9 ratio, was applied to the sections. The slides were incubated in the dark at 37°C for 60 min. The sections were processed for IF using the same protocol described previously. Briefly, the blocked sections were incubated with primary antibodies and corresponding secondary antibodies as mentioned above.

The cells were first permeabilized using 200 μL 0.1% Triton X-100 (ST795, Beyotime, Shanghai, China) for 15 min at room temperature. The cells were then covered with 200 μL TUNEL reaction mixture (enzyme solution: label solution = 1:9) and incubated in the dark at 37°C for 60 min. Nuclei were counterstained with DAPI (D106471, Aladdin, Shanghai, China) for 5 min. Staining results were visualized and assessed under a microscope (DP73, OLYMPUS, Japan).

**Co-IP**

The cells were lysed using RIPA lysis buffer (PR20001, Proteintech, Wuhan, China) supplemented with 1% protease inhibitor. The lysates were incubated on ice for 30 min and inverted several times at 10-min intervals during this period. The samples were centrifuged at 10,000 g for 3 min at 4°C, and the supernatant was collected. The protein concentration in the cell lysates was determined using a BCA protein assay kit (PK10026, Proteintech, Wuhan, China). The ELAVL1 rabbit antibody (11910-1-AP, Proteintech, Wuhan, China) was immobilized onto AminoLink coupling resin. The cell lysates were then added to the antibody-conjugated resin and incubated with gentle shaking at room temperature for 2 h. After incubation, the resin was washed with 200 µL IP lysis buffer, followed by centrifugation and additional washing steps. The immunoprecipitated proteins were subsequently analyzed by western blot as described below. The primary antibodies were as follows: TRIM2 mouse antibody (1:20000, 67342-1-Ig, Proteintech, Wuhan, China), ELAVL1 rabbit antibody (1:8000, 11910-1-AP, Proteintech, Wuhan, China), ubiquitin rabbit antibody (1:3000, ab134953, Abcam, UK) or β-tubulin rabbit antibody (1: 8000, 10094-1-AP, Proteintech, Wuhan, China). β-tubulin antibody was used as an internal control. The corresponding secondary antibodies were HRP-goat anti-mouse IgG (1:10000, SA00001-1, Proteintech, Wuhan, China) or HRP-goat anti-rabbit IgG (1:10000, SA00001-2, Proteintech, Wuhan, China). The whole cell lysates were used as a positive control. Finally, the ultrasensitive ECL detection kit (PK10003, Proteintech, Wuhan, China) was employed to visualize and analyze the proteins.

**Malondialdehyde (MDA) and superoxide dismutase (SOD)**

The SNc tissue samples were homogenized mechanically in a nine-fold volume of physiological saline (tissue weight: volume = 1:9) using an ice-water bath. The homogenates were subsequently centrifuged at 2500 r for 10 min, and the supernatant was collected for subsequent analysis. For cell samples, the cells were subjected to ultrasonic disruption, after which the lysates were used for the assays. Protein concentrations were quantified using a BCA protein concentration assay kit (BL521S, Biosharp, Beijing, China), with BSA as the standard. Next, MDA content and SOD activity were determined by MDA assay kit (A003, Nanjing Jiancheng Bioengineering Institute, Jiangsu, China) and SOD assay kit (A001, Nanjing Jiancheng Bioengineering Institute, Jiangsu, China).

**Real-time PCR**

Total RNA was extracted from SNc tissues using AG RNAex Pro RNA Extraction reagent (AG21101, Accurate Biology, Hunan, China). Reverse transcription was performed with HP All-in-one qRT Master Mix (RT203, YoungGen, Yunnan, China).

Real-time PCR was carried out using SYBR Green (SR4110, Solarbio, Beijing, China) and 2×Fast Taq plus Master Mix (BL1014, Biosharp, Anhui, China). Each 20 μL reaction contained 1 μL cDNA, 0.5 μL each of forward and reverse primers (10 μM), 0.5 μL SYBR Green, and 10 μL Master Mix. The cycling conditions were: 95 °C for 5 min, followed by 40 cycles of 95 °C for 10 s, 60 °C for 10 s, and 72 °C for 15 s, with a melting curve analysis from 60 °C to 94 °C. The primer sequences used were: ELAVL1 (forward: 5’-CAATCCCAACCAGAACA-3’, reverse: 5’-CACCAGAAATCCCACTCA-3’) and β-actin (forward: 5’-CTCCCTGGAGAAGAGCTATGA-3’, reverse: 5’-AGGAAGGAAGGCTGGAAGA-3’) were used. Relative expression was calculated by the 2^⁻ΔΔCt^ method.

**Western blot**

The SNc or the BE (2)-M17 cells were lysed with RIPA lysis buffer (PR20001, Proteintech, Wuhan, China) with 1% protease inhibitor (PR20032, Proteintech, Wuhan, China), followed by a 30-min incubation on ice. During this period, the samples were mixed upside down every 10 min. Then, the samples were centrifuged at 4℃ for 3 min at 10000 g and the supernatant was collected. BCA protein assay kit (PK10026, Proteintech, Wuhan, China) was used to determine the protein concentration. The protein received sodium dodecyl sulfate-polyacrylamide gel electrophoresis (SDS-PAGE) using 5% concentration gel, 10% and 13% separation gel. Subsequently, the protein was transferred onto a 0.45 μm polyvinylidene fluoride (PVDF) membrane (LC2005, Thermo Fisher Scientific, USA). The membrane was blocked using 5% skim milk, and then incubated overnight at 4℃ with TRIM2 mouse antibody (1: 20000, 67342-1-Ig, Proteintech, Wuhan, China), ELAVL1 rabbit antibody (1: 8000, 11910-1-AP, Proteintech, Wuhan, China), Bax rabbit antibody (1: 5000, 50599-2-Ig, Proteintech, Wuhan, China), Bcl-2 rabbit antibody (1: 2000, A19693, ABclonal, Wuhan, China) and β-tubulin rabbit antibody (1: 8000, 10094-1-AP, Proteintech, Wuhan, China). β-tubulin antibody was used as a control. The membrane was then incubated at room temperature for 1 h with HRP-conjugated goat anti-mouse IgG (1: 10000, SA00001-1, Proteintech, Wuhan, China) and HRP-conjugated goat anti-rabbit IgG (1: 10000, SA00001-2, Proteintech, Wuhan, China). The protein levels were visualized using the ultrasensitive ECL detection kit (PK10003, Proteintech, Wuhan, China).

**Statistical analysis**

All data were analyzed by GraphPad Prism software (version 9.5). Results were expressed as Mean ± SD. Normality of distribution was assessed using the Normality and Lognormality Tests function in GraphPad Prism, which includes the Shapiro-Wilk test. The *p* > 0.05 was considered consistent with a normal distribution. If both groups passed normality and had equal variances (F‑test), an unpaired two‑tailed Student’s t‑test was used. If variances were unequal, an unpaired t‑test with Welch’s correction was applied. If either group failed normality, the Mann-Whitney U test was used. For multi‑group comparisons (three or more groups), when all groups passed normality and variances were homogeneous (Brown-Forsythe test), one‑way ANOVA followed by Tukey’s test was performed. If normality failed or variances were unequal, the Kruskal-Wallis test followed by Dunn’s multiple comparisons test was used. In addition, Pearson correlation coefficient was used to evaluate the linear relationship between TRIM2 and ELAVL1 expression in human PD samples. Normality of both variables was confirmed before applying Pearson correlation. The p < 0.05 was considered as significant.

**Figure S1****.** Gene Ontology (GO) and Kyoto Encyclopedia of Genes and Genomes (KEGG) pathway enrichment analysis of the differentially expressed proteins (Fold Change > 1.5 or < 0.67, p < 0.05) on the basis of proteomic sequencing. (A) Top 15 significant enriched GO pathway terms, containing biological process (BP), cellular component (CC) and molecular function (MF), as well as top 15 significant enriched KEGG pathway terms was presented. The pathways related to neurons were highlighted in red.

**Figure S2.** TRIM2 reduced ELAVL1 expression in the PD rat SNc. (A) Real time PCR analysis of ELAVL1 mRNA levels in the rat SNc. (B) ELAVL1 protein expression in the SNc. (C) Double IF staining for TRIM2 (red) and ELAVL1 (green) in the SNc. Nuclei were counterstained with DAPI (blue). 400×, scale bar = 50 μm. (D) Double IF staining for TH (red) and ELAVL1 (green) in the SNc. Nuclei were counterstained with DAPI (blue). 400×, scale bar = 50 μm. n=6 per group. **p < 0.01, ****p < 0.0001.

**Figure S3.** TRIM2 or ELAVL1 expression in astrocytes and microglia of the rat SNc. (A) Double IF staining for TRIM2 (green) together with GFAP (astrocytes, red) or Iba1 (microglia, red) in the SNc. Nuclei were counterstained with DAPI (blue). 400×, scale bar = 50 μm. (B) Double IF staining for ELAVL1 (green) with GFAP (red) or Iba1 (red) in the SNc. Nuclei were counterstained with DAPI (blue). 400×, scale bar = 50 μm.

**Figure S4.** Expression of ELAVL1 and correlation between ELAVL1 and TRIM2 expression in human PD substantia nigra based on the GSE7621 dataset. (A) Upregulated ELAVL1 expression in the substantia nigra tissues from 16 postmortem human brain of PD patients compared to 9 healthy controls. (B) Pearson correlation analysis between TRIM2 and ELAVL1 expression values in the dataset (r = -0.4244, *p* < 0.05). **p < 0.01.

**References**

1.	Björklund T, Carlsson T, Cederfjäll EA, Carta M, Kirik D. Optimized adeno-associated viral vector-mediated striatal DOPA delivery restores sensorimotor function and prevents dyskinesias in a model of advanced Parkinson's disease. *Brain : a journal of neurology.* 2010;133(Pt 2):496-511.

2.	Perese DA, Ulman J, Viola J, Ewing SE, Bankiewicz KS. A 6-hydroxydopamine-induced selective parkinsonian rat model. *Brain research.* 1989;494(2):285-293.

3.	Chen W, Qiao D, Liu X, Shi K. Treadmill Exercise Improves Motor Dysfunction and Hyperactivity of the Corticostriatal Glutamatergic Pathway in Rats with 6-OHDA-Induced Parkinson's Disease. *Neural plasticity.* 2017;2017:2583910.

4.	Narbute K, Piļipenko V, Pupure J, et al. Intranasal Administration of Extracellular Vesicles Derived from Human Teeth Stem Cells Improves Motor Symptoms and Normalizes Tyrosine Hydroxylase Expression in the Substantia Nigra and Striatum of the 6-Hydroxydopamine-Treated Rats. *Stem cells translational medicine.* 2019;8(5):490-499.

5.	Olsson M, Nikkhah G, Bentlage C, Björklund A. Forelimb akinesia in the rat Parkinson model: differential effects of dopamine agonists and nigral transplants as assessed by a new stepping test. *The Journal of neuroscience : the official journal of the Society for Neuroscience.* 1995;15(5 Pt 2):3863-3875.

6.	Marie A, Leroy J, Darricau M, et al. Preventive Vitamin A Supplementation Improves Striatal Function in 6-Hydroxydopamine Hemiparkinsonian Rats. *Frontiers in nutrition.* 2022;9:811843.
